# The Effect of Individual and Neighborhood Socioeconomic Status on Gastric Cancer Survival

**DOI:** 10.1371/journal.pone.0089655

**Published:** 2014-02-25

**Authors:** Chin-Chia Wu, Ta-Wen Hsu, Chun-Ming Chang, Chia-Hui Yu, Yuh-Feng Wang, Ching-Chih Lee

**Affiliations:** 1 Department of Surgery, Dalin Tzu Chi Hospital, Buddhist Tzu Chi Medical Foundation, Chiayi, Taiwan; 2 Department of Otolaryngology, Dalin Tzu Chi Hospital, Buddhist Tzu Chi Medical Foundation, Chiayi, Taiwan; 3 Department of Nuclear Medicine, Dalin Tzu Chi Hospital, Buddhist Tzu Chi Medical Foundation, Chiayi, Taiwan; 4 Center for Clinical Epidemiology and Biostatistics, Dalin Tzu Chi Hospital, Buddhist Tzu Chi Medical Foundation, Chiayi, Taiwan; 5 Department of Education, Dalin Tzu Chi Hospital, Buddhist Tzu Chi Medical Foundation, Chiayi, Taiwan; 6 Department of Research, Dalin Tzu Chi Hospital, Buddhist Tzu Chi Medical Foundation, Chiayi, Taiwan; 7 School of Medicine, Tzu Chi University, Hualien, Taiwan; 8 Community Medicine Research Center and Institute of Public Health, National Yang-Ming University, Taipei, Taiwan; 9 Cancer center, Dalin Tzu Chi Hospital, Buddhist Tzu Chi Medical Foundation, Chiayi, Taiwan; University of Rochester, United States of America

## Abstract

**Purpose:**

Gastric cancer is a leading cause of death, particularly in the developing world. The literature reports individual socioeconomic status (SES) or neighborhood SES as related to survival, but the effect of both has not been studied. This study investigated the effect of individual and neighborhood SES simultaneously on mortality in gastric cancer patients in Taiwan.

**Materials and Methods:**

A study was conducted of 3,396 patients diagnosed with gastric cancer between 2002 and 2006. Each patient was followed for five years or until death. Individual SES was defined by income-related insurance premium (low, moderate, and high). Neighborhood SES was based on household income dichotomized into advantaged and disadvantaged areas. Multilevel logistic regression model was used to compare survival rates by SES group after adjusting for possible confounding factors.

**Results:**

In patients younger than 65 years, 5-year overall survival rates were lowest for those with low individual SES. After adjusting for patient characteristics (age, gender, Charlson Comorbidity Index Score), gastric cancer patients with high individual SES had 68% risk reduction of mortality (adjusted odds ratio [OR] of mortality, 0.32; 95% confidence interval [CI], 0.17–0.61). Patients aged 65 and above had no statistically significant difference in mortality rates by individual SES group. Different neighborhood SES did not statistically differ in the survival rates.

**Conclusion:**

Gastric cancer patients aged less than 65 years old with low individual SES have higher risk of mortality, even under an universal healthcare system. Public health strategies, education and welfare policies should seek to correct the inequality in gastric cancer survival, especially in those with lower individual SES.

## Introduction

Gastric cancer is a leading cause of death worldwide, with the 989,600 new cases in 2008 accounting for 8% of cancer cases. The 738,000 gastric cancer-related deaths worldwide in 2008 represented 10% of cancer deaths. In developing countries, more than 70% of new diagnoses and deaths from cancer are in gastric cancer patients [1]. In Taiwan and other Asian countries, gastric cancer remains an important cancer with high mortality. Gastric cancer ranks as the sixth-highest cause of cancer-related deaths in Taiwan, with a mortality rate of 6.8 per 100 000 [2].

According to the literature, gastric cancer incidence and survival are related to many risk factors, including individual and social risk factors. Individual factors include lymph node status, sex, race, genetics, individual socioeconomic status (SES) and diet; social factors include public health policies, availability of refrigeration and neighborhood SES [3]–[5]. The survival of patients with gastric cancer is related to individual SES [6], [7]. Patients in neighborhoods with the highest levels of SES may also enjoy better long-term survival [4].

Chang et al. pointed out that cancer patients with low individual SES who lived in disadvantaged neighborhoods had a higher risk of mortality than those in more favorable circumstances [8], including those with lung cancer, colorectal cancer, breast cancer, cervical cancer, prostate cancer, head and neck cancer, and pancreas cancer. We will consider the effect of neighborhood and individual SES on gastric cancer survival simultaneously in patients covered under the Taiwan National Health Insurance (NHI) system.

## Materials and Methods

### Ethics statement

This study was approved by the Institutional Review Board of Buddhist Dalin Tzu Chi General Hospital, Taiwan. Review board requirements for written informed consent were waived because all personal identifying information was removed from the dataset prior to analysis.

### Database

In March 1995, the Taiwan Department of Health integrated 13 health insurance schemes into a universal insurance program. As a compulsory social insurance program, the program covers approximately 99% of the residents of Taiwan and has contracts with 97% of medical providers [9]. Taiwan's NHI has the unique characteristics of universal insurance coverage and a single-payer system with the government as sole insurer. Patients have free access to seek care with any physician or hospital they choose. The insurance premium is calculated by the insurant's individual monthly income reported to the Bureau. The data for this study were collected from Taiwan's National Health Insurance Research Database (NHIRD) for the years 2002 to 2006. This dataset is organized and managed by Taiwan's National Health Research Institutes but collected by Taiwan's NHI Program. These databases were monitored for completeness and accuracy by Taiwan's Department of Health. To verify accuracy of diagnosis, Taiwan's NHI Bureau randomly reviews the charts of one per 100 ambulatory and one per 20 inpatient claims and conducts interviews of patients [10], [11].

Our study cohort consisted of Taiwan's incidental gastric cancer patients (International Classification of Diseases, Ninth Revision, Clinical Modification [ICD-9-CM] codes 147.9) who received some combination of surgery, adjuvant chemotherapy and chemoradiotherapy for their disease between 2002 and 2006. In Taiwan, the stage was not available in NHIRD. We selected the patients who received gastric resection and lymph nodes dissection as curative intent resection and tried to research the outcome of the gastric cancer patients.

### Measurement

The key dependent variable of interest was 5-year overall survival rate. We did not attempt to determine the cause-specific survival rate because the registry data we used did not contain this information. The use of overall survival data should not interfere significantly with our results because, as Roohan et al. have shown in a study adapting a clinical morbidity index for use with ICD-9-CM administrative databases, the survival models for all-cause mortality and cancer-specific mortality do not differ significantly [12].

The key independent variables of the current study were the effects on survival of individual SES and neighborhood SES. Survival of each gastric cancer patient was determined by linking that patient's 2002-2006 mortality data with claims data for the first curative treatment up to five years prior to death. Patient characteristics included age, gender, geographic location, treatment modality, comorbiditye and monthly income. Comorbidity was based on the modified Charlson Comorbidity Index Score (CCIS), a widely accepted measure for risk adjustment in administrative claims data sets [13].

### Individual-level measures

National health insurance in Taiwan identified each person's income and definite the level of insurance premium. We used the income-related insurance premium as a proxy for individual SES, an important prognostic factor for cancer [14]–[16]. The gastric cancer patients were classified into three groups: (1) low SES, lower than US$528 per month (New Taiwan Dollars (NT) $0 to $15,840), (2) moderate SES, between US$528 to $833 per month (NT $15,841 to $25,000), and (3) high SES, US$833 per month (NT $25,001) or more [17], [18]. We selected NT$15,840 as the low income level cutoff point because this was the government-stipulated minimum wage for full-time employees in Taiwan in 2006.

### Neighborhood-level socioeconomic status

Low neighborhood income was associated with health disparity [19]. We use neighborhood income as a proxy of neighborhood SES was based on the average neighborhood household income reported in Taiwan's 2001 Census. In that census, neighborhood household income was measured by township using per capita income (in New Taiwan dollars, NT$) based on 2001 tax statistics released by Taiwan's Ministry of Finance (http://www.fdc.gov.tw/dp.asp?mp=5). The categorization into advantaged or disadvantaged neighborhoods was based on the median values, with advantaged neighborhoods having a higher-than-median neighborhood household income and disadvantaged neighborhoods having a lower-than-median household income [20], [21].

### Other variables

We used population density, percentage of residents with college level or more education, percentage of residents ≥65 years old, percentage of residents who were agriculture workers, and the number of physicians per 100,000 persons to categorize residences into one of seven levels of urbanization, as previously described [22]. Urban residences were categorized as level 1, suburban residences were categorized as levels 2 and 3 and rural residences were categorized into levels 4 to 7. Hospitals were categorized by hospital accreditation level (medical center, regional hospital or district hospital). The geographic regions were recorded as northern, central, southern and eastern Taiwan.

### Statistical analysis

All statistical operations were performed using SPSS (version 15, SPSS Inc., Chicago, IL). Pearson's chi-square test was used for categorical variables such as gender, level of urbanization, geographic region of residence, CCIS, treatment modality, tumor extent and hospital characteristics (teaching level, ownership and caseload). Continuous variables were analyzed by one-way analysis of variance. The mortality rates between different SES was compared using Pearson's chi-square test.

The multilevel logistic regression model was used to analyze the relationship between the main outcomes of the different SES groups and those of the reference group after adjusting for hospital, and patient demographics age, gender, CCIS, urbanization and area of residence, adjuvant treatment modality (radiotherapy, chemotherapy, chemoradiotherapy) and hospital characteristics. In this study, the multilevel logistic regression method was used because of concern for the potential clustering effect in a hospital. A hospital-level random effect might account for possible correlations between hospitalization costs within a hospital's panel simply because of hospital policies, procedures, or physician compensation mechanisms that were unique to that hospital. A two-sided P-value (P<0.05) was considered significant.

## Results

### Demographic data and clinical characteristics

A total of 3396 gastric cancer patients who received curative-intent surgery with or without adjuvant therapy were included in this study (Table 1). Compared to those with high individual SES, gastric cancer patients with low individual SES were more likely to be older, to live in rural areas. There was no statistically significant difference in the comorbidities, geographic regions and treatment in regional and district hospitals between each individual SES groups.

**Table 1:** Baseline characteristics (gastric cancer with surgery, *n* = 3396).

Variables	Age <65 years (*n* = 1498)							Age ≧65 years (*n* = 1898)						
	High SES		Moderate SES		Low SES		P value	High SES		Moderate SES		Low SES		P value
	(*n* = 515)		(*n* = 509)		(*n* = 474)			(*n* = 59)		(*n* = 660)		(*n* = 1179)		
Mean age, years (±SD)	50.2±8.3		51.9±8.9		53.9±9.1		<0.001	73.2±6.7		74.2±5.5		75.2±5.9		<0.001
Gender							<0.001							<0.001
Male (%)	361	(70.1)	292	(57.4)	244	(51.5)		10	(16.9)	244	(37.0)	355	(30.1)	
Female (%)Comorbidities	154	(29.9)	217	(42.6)	230	(48.5)		49	(83.1)	416	(63.0)	824	(69.9)	
Chronic renal failure	2	(0.4)	7	(1.4)	3	(0.6)	0.184	0	(0.0)	9	(1.4)	10	(0.8)	0.417
Chronic obstructive pulmonary disease	3	(0.6)	3	(0.6)	1	(0.2)	0.613	2	(3.4)	20	(3.0)	42	(3.6)	0.832
Heart disease	6	(1.2)	1	(0.2)	2	(0.4)	0.111	4	(6.8)	24	(3.6)	58	(4.9)	0.313
Hypertensive	47	(9.1)	46	(9.0)	45	(9.5)	0.967	18	(30.5)	112	(17.0)	299	(25.4)	<0.001
Stroke	0	(0.0)	0	(0.0)	1	(0.2)	0.339	0	(0.0)	0	(0.0)	1	(0.1)	0.737
Diabetes mellitus	30	(5.8)	43	(8.4)	39	(8.2)	0.211	14	(23.7)	79	(12.0)	169	(14.3)	0.030
Adjuvant Therapy							0.221							0.952
Nil (%)	328	(63.7)	313	(61.5)	286	(60.3)		50	(84.7)	535	(81.1)	969	(82.2)	
Radiotherapy (%)	9	(1.7)	8	(1.6)	15	(3.2)		1	(1.7)	13	(2.0)	25	(2.1)	
Chemotherapy (%)	118	(22.9)	139	(27.3)	113	(23.8)		6	(10.2)	93	(14.1)	147	(12.5)	
Chemoradiotherapy (%)	60	(11.7)	49	(9.6)	60	12.7()		2	(3.4)	19	(2.9)	38	(3.2)	
Hospital characteristics							0.051							0.123
Teaching level														
Medical center (%)	374	(72.6)	334	(65.6)	331	(69.8)		45	(76.3)	419	(63.5)	808	(68.5)	
Regional (%)	137	(26.6)	169	(33.2)	133	(28.1)		13	(22.0)	223	(33.8)	344	(29.2)	
District (%)	4	(0.8)	6	(1.2)	10	(2.1)		1	(1.7)	18	(2.7)	27	(2.3)	
Urbanization							<0.001							<0.001
Urban (%)	200	(38.8)	111	(21.8)	146	(30.8)		25	(42.4)	34	(5.2)	427	(36.2)	
Suburban (%)	233	(45.2)	206	(40.5)	221	(46.6)		27	(45.8)	161	(24.4)	558	(47.3)	
Rural (%)	82	(15.9)	192	(37.7)	107	(22.6)		7	(11.9)	465	(70.5)	194	(16.5)	
Geographic Region							<0.001							<0.001
Northern (%)	302	(58.6)	217	(42.6)	287	(60.5)		32	(54.2)	215	(32.6)	757	(64.2)	
Central (%)	56	(10.9)	68	(13.4)	65	(13.7)		7	(11.9)	115	(17.4)	104	(8.8)	
Southern/ Eastern (%)	157	(30.5)	224	(44.0)	122	(25.7)		20	(33.9)	330	(50.0)	318	(27.0)	

Abbreviation: SES, socioeconomic status.

### Univariate survival analysis

**Figure 1:**
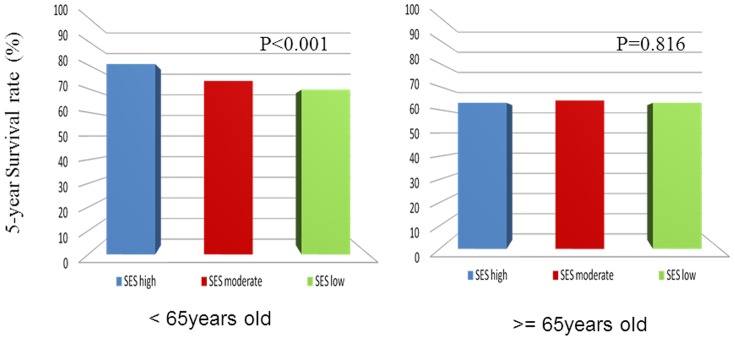
The effect of individual SES on survival rates in gastric cancer patients aged less than 65 years (1a) and aged 65 and above (1b).

**Figure 2:**
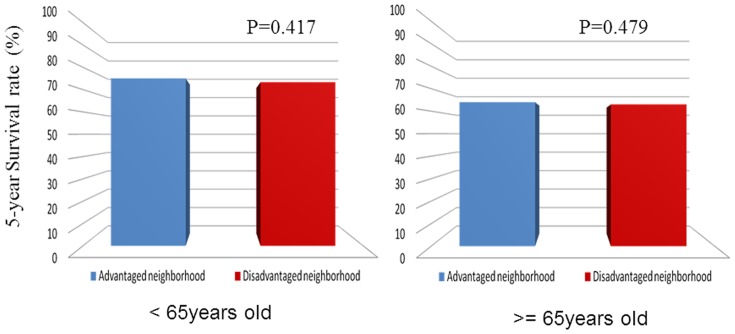
The effect of neighborhood SES on survival rates in gastric cancer patients aged less than 65 years old (2a) and aged 65 and above (2b).

As can be seen in Table 2, among gastric cancer patients younger than 65 years, those categorized as high individual SES had significantly better survival rates than all comparison groups (*P*<0.001) (Fig 1a). For those 65 years and above, individual SES was not statistically associated to gastric cancer survival in those who received curative-intent treatment (Fig 1b). There was no statistically significant difference in gastric cancer survival between different neighborhood SES for both age groups (Fig 2a and 2b).

**Table 2:** 5-year survival rate in different SES groups (Gastric cancer with curative surgery, n = 3396).

	Age <65 years (*n* = 1498)			Age ≧65 years (*n* = 1898)		
	*n*	Alive (%)	P value	*n*	Alive (%)	P value
Individual socioeconomic status.			<0.001			0.816
High	515	414(80.4)		59	37(62.7)	
Moderate	509	373(73.3)		660	424(64.2)	
Low	474	330(69.6)		1179	740(62.8)	
Neighborhood socioeconomic status			0.417			0.479
Advantaged	907	683(75.3)		957	613(64.1)	
Disadvantaged	591	434(73.4)		941	588(62.5)	

### Multilevel logistic regression model

The different medical provider was managed as a random intercept due to those patients might be treated in different providers with variant medical resources, capabilities, policies and physicians. A multilevel random intercept logistic regression model with the random effect of hospital showed that in high individual SES patients less than 65 years old, there was 68% risk reduction compared with those with lowest individual SES (adjusted odds ratio [OR]: 0.32; 95% CI: 0.17–0.61). Different neighborhood didn't seem to have significant effect on survival (Table 3). In aged patients, there was no statistical difference between those with different individual SES or those lived in different neighborhood (Table 3).

**Table 3:** Adjusted odds ratios of individual SES and neighborhood SES for mortality (gastric cancer with curative surgery, *n* = 3396).

Variable	Age <65 years			Age≧65years		
	Adjusted OR**	95%CI*	P value	Adjusted OR**	95%CI*	P value
Individual socioeconomic status						
Low	1			1		
Moderate	0.72	0.40–1.29	0.278	0.90	0.61–1.33	0.624
High	0.32	0.17–0.61	<0.001	1.17	0.49–2.80	0.713
Neighborhood socioeconomic status						
Disadvantaged	1			1		
Advantaged	0.61	0.33–1.10	0.101	0.71	0.48–1.05	0.089

*Abbreviation: 95% CI, 95% confidence interval.

**Adjust for the patients' age, gender, adjuvant therapy, urbanization, geographic region, comorbidities, and hospital characteristics.

## Discussion

This study found that, among gastric cancer patients in Taiwan younger than 65 years, those with high individual SES had a 68% lower risk of mortality than those with low SES, after adjusting for age at diagnosis, gender, comorbidities and hospital characteristics. The effect of SES was less evident in those 65 years and older. This study shows that the high individual SES lead to better survival of gastric cancer patients, even under a national health welfare and insurance system.

Previous studies analyzed SES status at either the individual or neighborhood level. Neighborhood socioeconomic context may affect health outcomes, after adjusting for individual SES [23]. Individual SES was reported to be related to survival independently of other factors, although the association was small [16], [24]. Kuwahara et al. reported a disparity in survival by occupation among gastric cancer patients in Japan, largely due to more advanced disease among the unemployed and manual laborers [25]. Patients with higher individual SES and therefore higher income may receive organized and opportunistic screening more often than those in lower income groups, thus permitting earlier detection [26]. In this study, we found that gastric cancer patients aged less than 65 years with low SES leads to the worst outcome, even in the patients under curative-intent therapy.

Deprived neighborhoods may indicate fewer medical resources, a more polluted environment, less social support and a poorer attitude toward health. In 1956, Torgersen et al. found that the prognosis for gastric cancer was related to the region of Oslo in which patients lived. Gastric cancer patients who lived in substandard housing areas had higher mortality rates [27], [28]. Fifty-six years later, Siemerink et al. used postal codes in The Netherlands to determine that neighborhood SES is an independent prognostic factor for gastric cancer survival [29]. In England, gastric cancer patients with lower neighborhood SES received gastrectomy, with no obvious association between survival and neighborhood SES [7], a hint that adequate treatment leads to similar survival rates in all patients. At the population level, disadvantaged SES neighborhoods may indicate inequities of medical resources, such as fewer hospitals and surgeons, which has been reported to impair disease treatment outcomes [30], [31].

Early diagnosis and multimodal treatment of gastric cancer improves outcomes, but overall mortality differs between rich and poor neighborhood [6]. Socioeconomic inequality is an independent factor influencing the prognosis of gastric cancer patients. Boyd reported that in Canada, the magnitude of the association between community income and survival would be weaker in Canada than in the U.S., because Ontario's universal, comprehensive, provincial health system might mitigate the adverse impact of poverty on cancer outcome by removing barriers to care for the poor [32]. To compare with neighborhood and individual effect in our study, the patients aged less than 65 years with high individual SES had the best survival. We also found that the difference was not statistically significant in community-income represented neighborhood SES. In patients older than 65 years, neither individual SES nor neighborhood SES indicates to different survival outcomes. Such observations indicate that, even under an universal health-insurance system, the patient with low individual SES has the worst survival rate of all patients. Gastric cancer patients need early detection and multimodal treatment to improve their outcomes. Patients with higher SES communicate more effectively with medical profession during the receipt of health care [33]. Patients living in disadvantaged neighborhoods also tend to have higher levels of social isolation, depression and occasional stress than patients living in neighborhoods with high SES [34].

This study has several limitations. First, the diagnosis of gastric cancer, as well as other comorbidities in this study, was garnered from ICD-9-CM codes on NHI claims. While this method of identification is not ideal, the NHI Bureau in Taiwan does randomly review the charts and interview patients to spot verify the accuracy of diagnosis. Another limitation was our lack of access to detailed information on gastric cancer stage, pattern of relapse and other risk factors, such as tobacco use and dietary habits. Curative and palliative treatment were also a limitation. Although we selected the patients with resection of stomach and lymph nodes dissection, exact extensiveness and type of dissection was not clear. However, given the robustness of the evidence, statistical analysis, and sensitivity analysis in this study, these limitations are unlikely to compromise our results.

Gastric cancer patients aged less than 65 years with low SES have poorer outcomes than those with high SES. For such patients, greater accessibility, education and information will likely improve their gastric cancer outcomes. Although the system of social welfare and national health insurance broke the health inequality between different neighborhoods SES, and provide for medical service to these low SES patients, the health gap associated with personal poverty remains a challenge.
